# The Transfer Characteristics of Potentially Toxic Trace Elements in Different Soil-Rice Systems and Their Quantitative Models in Southeastern China

**DOI:** 10.3390/ijerph16142503

**Published:** 2019-07-13

**Authors:** Zheyao Yu, Jiaqi Dong, Weijun Fu, Zhengqian Ye, Wanyi Li, Keli Zhao

**Affiliations:** 1 State Key Laboratory of Subtropical Silviculture, Zhejiang A&F University, Lin′an 311300, China; 2 Key Laboratory of Soil Contamination Bioremediation of Zhejiang Province, Zhejiang A&F University, Lin’an 311300, China; 3 Zhejiang Provincial Key Laboratory of Carbon Cycling in Forest Ecosystems and Carbon Sequestration, Zhejiang A&F University, Lin’an 311300, China; 4 The Bureau of Agriculture and Forestry, Luqiao District, Taizhou City 318050, China

**Keywords:** Soil-rice system, PTEs, Transfer model, Controlling factors, Enrichment index

## Abstract

The potentially toxic trace elements (PTEs) transfer characteristics in the soil-rice system plays an important role in soil quality management, and it can be used to guide the safe rice production. We collected soil and rice samples from three typical rice production areas (Nanxun, Shengzhou, Wenling in northern, central, and southern parts of Zhejiang Province, China). The controlling factors of PTEs′ transfer were studied for Hybrid rice and *Japonica* rice. The results indicated that the pH, organic matter (OM), and electrical conductivity (EC) values of Shengzhou were all lower than that of the other two production areas (Nanxun and Wenling). The concentrations of PTEs in the soils of Wenling were significantly higher than that in the other two areas, while the concentrations of PTEs in the rice of Shengzhou were significantly higher than that of Wenling and Nanxun (*p* < 0.05). The enrichment index (EI) of PTEs were also different in the three production areas. The EIs of Cd and Zn were higher than that of Cu and Ni in the three production areas, and the EIs in Shengzhou were significantly higher than that of other two areas (*p* < 0.05). The soil physico-chemical properties and PTEs’ fractions both played important roles in PTEs transfer in the soil-rice system. The log-linear model of EI for PTEs can predict the availability of PTEs in the soil-rice system under practical production areas. The accuracy of the model prediction of EI for *Japonica* rice was better than that for the Hybrid rice. The prediction model of Ni was better than that of other PTEs for both rices.

## 1. Introduction

Rice (*Oryza sativa* L.), is the most important agricultural crop in southern and southeastern Asia, feeding more than two billion people [1]. About 90% of the world′s rice is planted and consumed in these areas [2]. However, due to industrial activities (such as mining and smelting), the over-application of agro-chemicals and sewage sludge, and waste-water irrigation, excessive potentially toxic trace elements (PTEs) are accumulated in paddy soils [3,4,5]. The contamination of paddy soils with PTEs can cause an increase in the uptake of PTEs by rice. Once the concentrations of PTEs in rice grains exceeded the threshold value (such as cadmium: 0.2 mg kg^−1^, Lead: 0.2 mg kg^−1^ in China), they will pose long-term environmental and health implications [6,7]. The PTEs pollution in agricultural soils has caused increasing public concern for food security worldwide, due to their non-biodegradability and persistence [8,9,10].

The quality of rice greatly affects human health, as consuming rice with elevated PTEs can seriously deplete body stores of iron (Fe), vitamin C, and other essential nutrients, since rice is the staple food for a large part of the human population. For example, the intake of cadmium (Cd) by means of rice consumption is the main exposure pathway for human to Cd [1]. The residents of an irrigation area in Dayu, Jiangxi Province of China, were found to suffer from Cd-related renal tubular dysfunction, with about 99.5% of the oral Cd contributed by rice and vegetable consumption [11]. It is reported that the annual amount of crops (mainly are rice) that were polluted by PTEs is 12,000,000 ton in China [12]. Therefore, it is important to protect paddy soils and ensure their sustainability. 

A lot of work has been carried out to study the PTEs transfer characteristics in soil-rice system [2,13,14,15]. The uptake of PTEs by rice depends not only on the total concentrations, but also on their mobility and fractions [16]. The chemical fractions of PTEs in soils can be defined as exchangeable, carbonate bound, organic bound, Fe-Mn oxide bound, and residual forms [17]. The chemical forms of metals in soils can vary strongly, depending on soil properties, such as pH, soil organic matter (SOM), cation exchange capacity (CEC), and soil texture [18,19]. In such a case, soil physico-chemical properties, rice genotypes, and their fractions all strongly influenced the PTEs transfer in soil-rice system. Generally, linear, exponential, and logarithmic models were applied to describe the transfer characteristics based on the above-mentioned soil variables (PTEs and soil pH, SOM, etc.). Differences of transfer models were found among the studies, which was probably related to different study areas and different soil properties characteristics.

However, most of the previous studies on PTEs transfer in soil-rice system were based on pot or field experiments [2,20], which could cause potential errors if such models were used to predict or guide practical PTEs′ transfer in rice production areas. Little information is available regarding the soil-rice system in practical production areas at the regional scale. In the field environment, there are spatial variation of soil physico-chemical properties, PTEs concentrations, and rice plants growth. It is necessary to carry out the research that is related to the PTEs transfer model under the real rice growth environment. Our models could be better for further guiding the safe rice production when compared to the transfer models that were obtained from the pot experiment.

In this study, three rice production counties were chosen in the subtropical regions of China. The main objectives of this study were: (1) to characterize the status of PTEs status in soil and rice grains; (2) to investigate the impact factors of PTEs′ transfer at different production areas; and, (3) to build up the PTEs transfer models for soil-rice system.

## 2. Material and Methods

### 2.1. Study Area and Sampling Site Description

**Figure 1:**
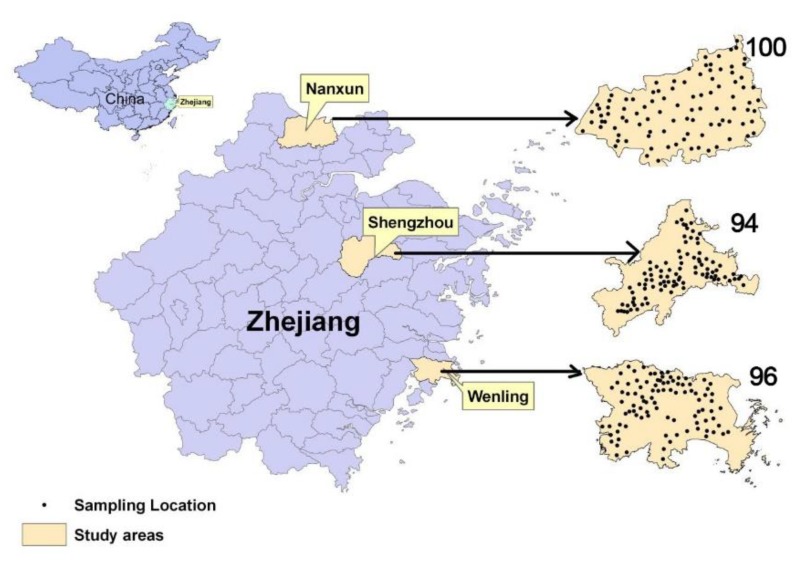
Location of the study areas and corresponding samples.

This research was carried out in the main rice production areas of Nanxun (120°04′ to 120°29′ E, 30°38′~30°56′ N, northern part of Zhejiang province), Shengzhou (120°28′~121°07′ E, 29°20′~29°50′ N, central part of Zhejiang), and Wenling (121°10′ to 121°44′ E, 28°13′ to 28°32′ N, southern part of Zhejiang), China (Figure 1). The agriculture in Zhejiang Province was well developed and rice was the main grain crop [21]. It is broadly representative of typical rice production management in southern China. The soil in the study area is a typical paddy soil, which has been used for aquatic rice production. The term “paddy soil” is related to land use, but not to any strict definition of soil type in the pedology [16]. The above three study areas all have a subtropical marine monsoon climate, with average annual rainfall of 1230–1693 mm and mean annual temperature of 15.7–17.3 °C. The main rice types are *Japonica* and Hybrid rice in Nanxun and Shengzhou, respectively. Both rice types are planted in Wenling.

During the rice harvest season, a total of 100, 94, and 96 pairs of rice grain and soil samples were taken from Nanxun, Shengzhou, and Wenling, respectively (Figure 1), by means of a randomized design, on the basis of a land use map at 1:50,000 scale. Overall, one pair of soil-rice sample was taken per kilometer square based on the rice planting area. Each sample was the composite of at least five sub-samples within a distance of 10 m surrounding a specific sampling location. The rice and their rooted soil samples (0–15 cm) were collected from each site. The samples of soil and rice weighted at 1 and 0.5 kg, respectively. The longitudes and latitudes of the sampling points were recorded while using a portable global positioning system (GPS) receiver. In this study, the collected samples were mainly distributed in the plain paddy fields. The mountainous and downtown areas were avoided due to rare rice production distribution.

### 2.2. Laboratory Analyses

The soil samples were air-dried in the laboratory and sieved to pass a 2 mm nylon mesh for soil chemical and physical analyses. A portion of the soil samples were ground in an agate mortar to pass through a 0.149 mm sieve and then stored in sealed polyethylene bags for further soil Cd, Cu, Pb, Zn, and Ni (PTEs) and soil organic matter analyses.

The soil properties were measured according to the standard methods in China [22]. Soil pH and electrical conductivity (EC) were determined in an aqueous suspension (1:2.5 and 1:5 soil-water ratio, respectively). Soil organic matter was analyzed by the potassium dichromate wet combustion procedure. Soil particle size distribution (sand, silt, and clay content) was analyzed while using the hydrometer method.

For PTEs analysis, the soil samples were digested while using strong acids of HF, HNO_3_, and HClO_4_. The total Cd concentration in soils was analyzed by graphite furnace atomic absorption spectroscopy (GFAAS, PerkinElmer AA800, Waltham, MA, USA), with the detection limit of 0.002 mg L^−1^. The total Cu, Ni, Pb, and Zn in soils were determined while using flame-atomic absorption spectroscopy (FAAS, PerkinElmer AA800, Waltham, MA, USA) with the detection limits of 0.05, 0.1, 0.2, and 0.05 mg L^−1^. The PTEs fractions in soils were analyzed while using the modified method of sequential extraction that was initially proposed by Tessier [23]. The extraction method operationally defines PTEs in five forms Wong [17]: (1) exchangeable form (1 M MgCl_2_ pH = 7.0), (2) carbonate bound form (1 M NaOAc pH = 5.0), (3) Fe-Mn oxide bound form (0.04 M NH_2_OH·HCl in 25% HOAc), (4) organic/sulfide bound form (0.02 M HNO_3_/30% H_2_O_2_ pH = 2.0/3.2 M NH_4_OAc in 20% HNO_3_), and (5) residual form (HF/HNO_3_/HClO_4_). The supernatant was separated by centrifugation at 3500× *g* for 20 min. after each sequential extraction and then Cd concentration was determined by GFAAS; other PTEs fractions were determined by FAAS. In this study, the carbonate bound form of PTEs in soils was below the limit of detection, which was probably related to the acidic soils in the study areas. The exchangeable form of Cu was also below the limit of detection. Thus the results of other forms were used for further analyses.

The rice grain samples were oven-dried at 105 °C for 1h, and then at 70 °C to constant weight. Hulls were removed from rice grain samples. Subsequently, the rice samples were ground to pass through a 0.149 mm sieve and then stored in sealed polyethylene bags. These samples were digested by HNO_3_ and H_2_O_2_. The Cd, Cu, Ni, and Pb concentrations in rice were determined by GFAAS (PerkinElmer AA800, Waltham, MA, USA), with the detection limits of 0.002, 0.014, 0.07, and 0.05 mg L^−1^, respectively. The Zn concentrations in rice were determined by FAAS with a detection limit of 0.05 mg L^−1^. 

The accuracy of determinations was verified while using the Chinese standardized reference materials (GSS-4 and GSS-15 for soil samples; GBW(E) 080684 for rice samples). The samples were measured in duplicate. The recovery of sequential extraction for PTEs was calculated in order to ensure the analytical quality during the sequential extraction analysis. The average recovery of sequential extraction for Cd, Cu, Ni, Pb, and Zn were 90.4%, 90.3%, 91.7%, 94.5%, and 91.2% with the standard deviations of 8.5%, 6.4%, 6.7%, 8.3%, and 6.8% when compared with the total concentration of PTEs in soils. Thus, the analytical quality was acceptable for sequential extraction [9].

### 2.3. Statistical Analyses

The significance of differences were tested while using analysis of variance (ANOVA) and Duncan’s Multiple Comparison test in SPSS (Version 19.0) [24,25]. The Duncan’s Multiple Comparison test was used to separate the means when the ANOVA analysis indicated a significant difference. An alpha level of 0.05 for significance was used in all statistical analyses, unless otherwise mentioned. The normality and homogeneity of raw data were tested before performing the ANOVA analysis, and the data were log-transformed if the homogeneity of the variance was not met [26,27,28,29,30,31]. Principal component analysis (PCA) was done while using normalized data in the SPSS software (Version 19). 

## 3. Results

### 3.1. Soil Physico-Chemical Properties of the Three Production Areas

The mean pH and SOM values of Shengzhou were lower than that of Nanxun and Wenling, respectively (Table 1). The electrical conductivity (EC), silt, and clay contents in Shengzhou were also lower than that of the other two areas, while the sand content in Shengzhou was significantly higher than that of Nanxun and Wenling (*p* < 0.05). The differences in soil properties could influence the enrichment index of PTEs in the three production areas.

### 3.2. The Total PTEs Concentrations in Soil and Rice

There were significant differences of total PTEs concentrations among the studied areas (Table 2). The total PTEs concentrations in soils of Shengzhou had relatively low mean values when compared to the other two areas. The mean Cd, Cu, and Zn concentrations in the soils of Wenling were significantly higher than that of Nanxun and Shengzhou, respectively (*p* < 0.05). No significant differences in concentrations of PTEs in soils were found between the Nanxun and Shengzhou areas.

The mean PTEs contents in rice decreased by the order of Shengzhou > Wenling > Nanxun (Table 2). The Cd, Cu, and Zn contents in rice of Shengzhou and Wenling were significantly higher than that of Nanxun, respectively (*p* < 0.05), while no significant differences of the Cd, Cu, and Zn contents in rice were found between Shengzhou and Wenling. The Ni in rice was significantly different among the studied areas.

Although the total PTEs in soils of Shengzhou were low, their corresponding concentrations in rice were high, which indicated that the bio-availability of PTEs in Shengzhou was high, and the total PTEs in soils could not effectively reflect the bio-availability of PTEs in the soil-rice system.

### 3.3. The PTEs’ Fractions Distribution in Soils

**Figure 2:**
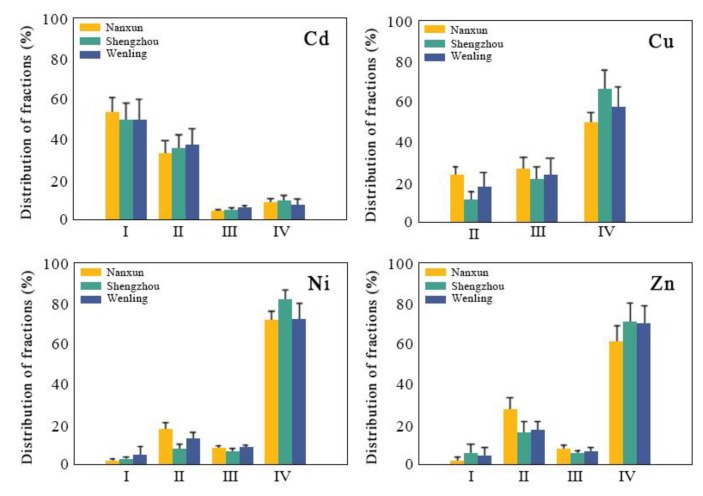
Distribution of PTEs′ fractions as percentages of the total concentrations in the soils of the study areas (I: exchangeable fraction; II: Fe-Mn oxide bound fraction; III: organic bound fraction; and, IV: residual fraction).

The percentage values of different fractions were used in order to compare the PTEs’ bio-availability of different production areas (Figure 2). For Cd in soils, the main component was the exchangeable fraction. The concentrations of fractions of Cd were in the order of exchangeable > Fe-Mn oxide bound > residual > organic bound. For Cu, Ni, and Zn in soils, the residual fraction was the main component, while the exchangeable fraction was low. Especially for Cu, its exchangeable fraction was lower than the detection limit. The concentrations of the fractions of Cu were in the order of residual > organic bound > Fe-Mn oxide bound, while soil Ni and Zn had similar fractions distribution with the order of residual > Fe-Mn oxide bound > organic bound > exchangeable.

### 3.4. The Enrichment Index in the Soil-Rice System

**Figure 3:**
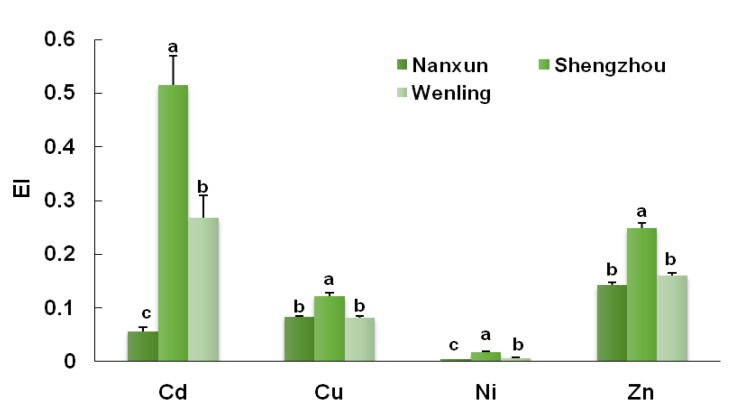
Enrichment index (EI) of PTEs in the soil-rice system. Different letters indicate significant differences of selected variables at 0.05 level.

The enrichment index (EI, the metal concentration in rice divided by that in soil) in the soil-rice system of Shenghzou and Wenling had similar distribution traits (Figure 3), with the highest values of 0.521 and 0.269 for Cd, respectively, followed by elements of Zn, Cu, and Ni. In the Nanxun area, Zn had the highest EI value (0.143), followed by Cu, Cd, and Ni.

There were significant differences among the EIs of the PTEs in the three study areas. The EIs in Shengzhou were significantly higher than the corresponding values in Nanxun and Wenling (*p* < 0.05). The EIs of Cd and Ni of Wenling also were significantly higher than that of Nanxun, while no significant differences were found for Zn and Cu in these two study areas. Therefore, the PTEs transfer ability decreased, as Shengzhou > Wenling > Nanxun.

### 3.5. The Impact Factors for the PTEs′ Transfer of the Soil-Rice System

Previous studies indicated that rice genotypes, PTEs in soils, and soil physico-chemical properties had a clear effect on the availability of PTEs in the soil-rice system [32]. The principle component analysis (PCA) was applied to analyze the correlation between EIs and other soil properties based on rice genotypes to determine the key impact factors. In this study, the Hybrid rice from Shengzhou and the *Japonica* rice from Nanxun were chosen.

Table 3 shows the PCA loading factors for Hybrid rice. The first PCA components of Cd included EIs, exchangeable fraction, Fe-Mn bound fraction, organic bound fraction, pH, and organic matter content (OM); the second components included the sand, silt, and clay content; the third component included EIs, residual fraction, OM, and electrical conductivity (EC). These results indicated that the EI of Cd of hybrid rice had a correlation with its fractions, pH, OM, and EC. Similar to Cd, the fractions, pH, OM, and EC were the main factors that influenced the Zn transfer in Hybrid rice, while EC was the only factor that was correlated with EIs of Cu and Ni. For the *Japonica* rice (Table 4), the Cd transfer was mainly influenced by Cd fractions, pH, OM, EC, and sand content. The pH, silt, and clay content were correlated with EI of Cu. The pH and exchangeable fractions were the two factors that were correlated with the EIs of Zn and Ni.

### 3.6. Predicted Model Chosen

The Hybrid rice in Shegnzhou and the *Japonica* rice in Nanxun were chosen to build up their corresponding transfer models. Additionally, both rice types in Wenling were applied to validate the accuracy of the models. The EI was used to stand for the PTEs transfer capability, while PTEs’ fractions and physico-chemical properties of soils were regarded as the impact factors. 

Based on previous studies [3,9,14,15], the linear, logarithmic model, and their combination model were tested (Table 5), while using PTEs’ fractions (exchangeable, Fe-Mn bound, organic bound, and residual fractions) and soil physico-chemical variables (pH, OM, EC, sand, silt, and clay). Model 1 was a logarithmic model; model 2 and 3 were a linear and logarithmic model with different combination (see the footnote of Table 5); model 4 was a linear model. Four different models were compared (Table 5). The linear and logarithmic model (Model 2) were chosen based on the highest coefficient of determination, which can be expressed as:

lnEI=alnI+blnII+clnIII+dlnIV+epH+fOM+gEC+hSand+iSilt+jClay+k

where EI is enrichment index; I, II, III, and IV stand for exchangeable form, Fe-Mn oxide bound form, organic bound form, and residual form of heavy metals; *a*, *b*, *c*……*k* are the regression coefficients of the model.

### 3.7. The Best Transfer Model Determination and Cross-Validation

Table 6 lists the regression coefficients for the regression model. The coefficients of determination (R) of the transfer models for PTEs were different. For the Cd of Hybrid rice, the R values that were caused by PTEs’ fractions and soil properties were 0.65 and 0.56, respectively, which reached an extremely significant level (*p* < 0.01). For the Cd of *Japonica* rice, the R values were 0.50 and 0.76, respectively, which also reached an extremely significant level (*p* < 0.01). A similar phenomenon was found for EI of Zn. For Hybrid rice, PTEs’ fractions and soil properties both had an extremely significant correlation with EI of Cu (*p* < 0.01). For *Japonica* rice, the soil properties had an extremely significant correlation with EI of Cu (*p* < 0.01), while PTEs’ fractions had significant correlation with EI of Cu (*p* < 0.05). For the EIs of Ni, its fraction had no significant correlation with the Ni of Hybrid rice (R = 0.20, *p* > 0.05), while the soil properties had an extremely significant correlation (*p* < 0.01). PTEs’ fractions and soil properties both had extremely significant correlation with the EI of Ni in *Japonica* rice. Therefore, the final regression coefficients were obtained to fit the best model 2 based on the PTEs’ fractions and the physico-chemical properties of soils (Table 7).

**Figure 4:**
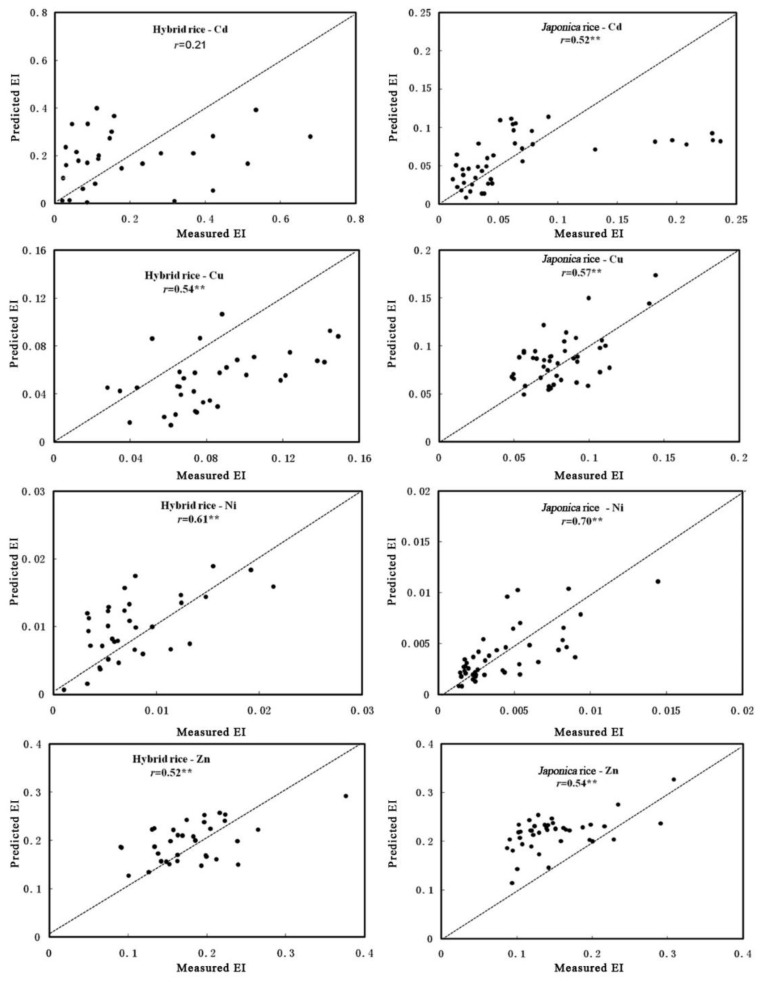
Measured and predicted EI values for PTEs for the soil-rice system of Wenling basing on the best transfer model. ** indicates a significant level of 0.01.

The data that were collected from Wenling were used to validate the prediction accuracy (Figure 4). Extremely significant correlations (*p* < 0.01) were found between measured value and predicted value of other PTEs for the Wenling study area, except for the Cd of Hybrid rice. For Cd, the correlation coefficient between the measured value and predicted value was only 0.21 (*p* > 0.05), which indicated that the accuracy of the transfer model was weak for EI of Cd of Hybrid rice. For *Japonica* rice, the correlation coefficient of Cd was 0.52 (*p* < 0.01). When the EI values of Cd were low, its predicted values tended to be higher than the measured values; when the EI values were high, its predicted values were lower than the measured values. A critical EI value of 0.2 for Hybrid rice and of 0.1 for *Japonica* rice was found. For the EIs of Cu, the correlations between measured and predicted values were extremely significant (*p* < 0.01). However, for the Hybrid rice, the predicted value of EIs of Cu was smaller than the measured values, the difference increased with the EI value increase. The transfer model of Zn had a similar prediction accuracy with Cu, even under the small EIs, both rices had higher predicted values than the measured values. Ni had the best prediction model with the highest correlation coefficients of 0.61 and 0.7 (*p* < 0.01).

## 4. Discussion

### 4.1. The Effects of Impact Factors On PTEs’ Transfer in the Oil-Rice System

There is a lot of information available with respect to the genotypes, environmental effects, and their interaction impact on the PTEs’ transfer of rice. Cheng [33] found that genotypes had a clear effect on Cd, followed by the interaction of genotype and environment. Zeng [20] selected 138 genotypes of rice to determine the Cd, Cr, and Pb uptake while using a pot experiment, and found that the interaction of genotype and environment played an important role in the PTEs’ uptake of rice. In this study, we selected the practical paddy fields in Nanxun, Shengzhou, and Wenling. The rice genotypes, environment condition, and agricultural management were different, which could cause the effect of interactions of genotype and the environment. Overall, two main rice genotypes (Hybrid and *Japonica* rice) were distributed in the three production areas. The main rice genotype in Nanxun was *Japonica* rice and Hybrid rice in Shengzhou. Both rice genotypes were found in Wenling. Therefore, the Hybrid rice could be an important factor that causes high transfer capability of PTEs in Shengzhou.

Soil physico-chemical properties also play an important role in PTEs’ transfer from soil to crops [13]. In Shengzhou, the low pH, SOC, and EC could promote PTEs’ transfer from soil to rice [21]. In Nanxun and Wenling, the relatively high pH, EC, silt, and clay contents weakened the accumulation of PTEs in rice. This is another reason for the high PTEs’ concentrations in rice; even their corresponding concentrations in soil were low in the Shengzhou area.

The EIs of Cd and Zn in the three production areas were higher than that of Cu and Ni, which indicated that the transfer ability and bio-availability of Cd and Zn in the soil-rice was relatively strong. The soil non-residual forms (exchangeable, Fe-Mn oxide bound, and organic bound, which had relatively high bioavailability) of Cd and Zn accounted for more than 90% of the total PTEs’ content. For example, the non-residual forms of Cd in Nanxun, Shengzhou, and Wenling accounted for 91.6%, 90.2%, and 92.8% of the total Cd in soil, respectively, while the non-residual forms of Ni in the above three studied areas accounted for 1.8%, 2.5%, and 4.7%. These results were in line with previous studies [34,35]. Zhu et al. found [34] that the Cd content in rice was not significantly correlated with total Cd in soil, but with exchangeable Cd in soil. Wang [35] reported that the EIs of Cd and Zn were significantly higher than other elements in paddy fields.

### 4.2. The Best PTEs’ Transfer Model for the Soil-Rice System

Previous studies indicated that the PTEs transfer models in the soil-crops system mainly included linear, exponential, and logarithmic models [3,36,37,38,39,40,41,42]. Among the above three models, the exponential and logarithmic models can be transformed into each other while using mathematics methods. Therefore, the models that were related to PTEs in soil-crops system can be linear and logarithmic models.

Wang et al. [43] applied a linear model to predict the Pb, Cu, and Ni content in rice and the logarithmic model to predict the Cd and Cr content in rice while using the PTEs and physical-chemical properties in soils. Zhang et al. [40] also used a single linear modle to predict the bioavailability of heavy metals in polluted soils to rice. Römkens et al. [3] took the logarithmic model to predict the Cd concentration in brown rice in Taiwan. In this study, the mixed model (linear and logarithmic model) was used to satisfactorily fit the relationship between the EIs and soil PTEs’ fractions and soil physico-chemical properties. Generally speaking, the soil physico-chemical properties model was better than that of the PTEs’ fractions model. Taking Ni as an example, both of the regression models using the soil physico-chemical properties reached an extremely significant level (Table 6), while the fitted model of Ni for Hybrid rice did not reach the significant level while using PTEs’ fractions in soil. This indicated that the driven effect caused by soil properties was stronger than that of PTEs’ fractions. When both PTEs’ fractions and soil properties were considered, their fitted models all reached an extremely significant level (*p* < 0.01).

Even the regression model reached the significance level, the *R* (Max.: 0.83, Table 6) value was relatively lower than that of the pot experiments and plot experiments [44,45,46,47]. This is mainly due to the complexity of an ecosystem under practical production with the spatial variation of soil properties, PTEs’ concentrations, and practical management. In this study, we focused on the PTEs’ fractions and soil properties (pH, SOM, EC, and particle size composition); however, other factors may also influence the PTEs’ transfer in the soil-rice system, such as the soil redox potential and soil phosphorus content [48,49,50]. The prediction accuracy may be improved if more factors were included in the model.

In this study, the transfer models were different for different rice types and PTEs. Such models could predict the EIs of soil-rice system at practical production areas, even if the accuracy of the model was weak. Overall, the prediction accuracy for *Japonica* was better than for Hybrid rice. However, Cd content was overestimated due to the transfer model. 

## 5. Conclusions

In this study, the transfer ability of PTEs in the soil-rice system was found to be in the order of Shengzhou > Wenling > Nanxun, which implies that the bio-availability of PTEs at the Shengzhou site where Hybird rice is grown was high. The bio-availability of Cd and Zn was significantly higher than that of Cu and Ni.

The physico-chemical properties, such as pH and EC and PTEs’ exchangeable fraction, were the main factors that affected the transfer of PTEs in the soil-rice system. Based on the genotype, the best fitted linear and logarithmic model could satisfactorily predict the PTEs’ EI, except the Cd content of Hybrid rice. Therefore, more work needs to be carried out to improve the transfer models.

## Figures and Tables

**Table 1:** The physico-chemical properties of the paddy soils in the study areas (Mean ± SD).

Study Area	pH	SOM (g·kg^−1^)	EC (μs·cm^−1^).	Sand (%)	Silt (%)	Clay (%)
Nanxun	6.15 ± 0.62a	41.0 ± 12.0a	385 ± 163a	1.08 ± 2.09c	73.87 ± 6.75a	21.78 ± 5.27b
Shengzhou	5.52 ± 0.63a	39.4 ± 9.6a	172 ± 11b	15.24 ± 12.30a	49.33 ± 13.61b	20.90 ± 4.64b
Wenling	5.73 ± 0.79a	43.3 ± 12.7a	254 ± 163b	4.89 ± 6.15b	62.10 ± 10.25ab	28.28 ± 5.52a

SOM, soil organic matter; EC, electrical conductivity. Different small letters mean a significant difference at 0.05 level.

**Table 2:** The total potentially toxic trace elements’ (PTEs’) concentrations of the paddy soils and rice in the study areas (Mean ± SD).

Study Area	Cd_soil_	Cu_soil_	Ni_soil_	Zn_soil_	Cd_rice_	Cu_rice_	Ni_rice_	Zn_rice_
Nanxun	0.21 ± 0.07b	31.06 ± 7.45b	32.14 ± 6.75a	106.82 ± 30.05b	0.011 ± 0.015b	2.49 ± 0.74b	0.125 ± 0.173c	14.28 ± 2.70b
Shengzhou	0.20 ± 0.09b	28.64 ± 13.36b	27.03 ± 22.04a	98.74 ± 32.06b	0.09 ± 0.10a	2.98 ± 1.08a	0.35 ± 0.28a	22.41 ± 3.54a
Wenling	0.31 ± 0.38a	41.13 ± 19.74a	33.89 ± 12.69a	137.03 ± 33.83a	0.072 ± 0.105a	3.09 ± 0.96a	0.22 ± 0.23b	20.69 ± 4.71a

Cd_soil_, Cd in soil; Cu_soil_, Cu in soil; Ni_soil_, Ni in soil; Zn_soil_, Zn in soil; Cd_rice_, Cd in rice; Cu_rice_, Cu in rice; Ni_rice_, Ni in rice; Zn_rice_, Zn in rice.

**Table 3:** The principal component analysis (PCA) results of soil environmental factors for Hybrid rice.

Item	Cd			Cu				Ni				Zn		
	PC1	PC2	PC3	PC1	PC2	PC3	PC4	PC1	PC2	PC3	PC4	PC1	PC2	PC3
EI	**−0.36**	0.10	**−0.49**	0.08	−0.17	**−0.85**	0.09	0.02	0.17	0.15	**−0.84**	**−0.54**	0.09	**0.57**
Exchangeable	**−0.92**	0.07	0.30	/	/	/	/	0.21	−0.14	**0.84**	−0.01	**0.62**	−0.13	**0.64**
Fe-Mn bound	**0.93**	−0.03	−0.16	−0.22	**0.84**	0.15	−0.28	**0.91**	0.06	−0.22	0.11	**0.86**	0.32	−0.08
Organic bound	**0.77**	−0.05	0.18	0.31	**0.82**	0.20	0.32	**0.78**	0.42	0.14	0.14	**0.58**	**0.52**	0.17
Residual	0.15	−0.11	**−0.80**	−0.14	**−0.95**	−0.22	−0.12	**−0.94**	−0.16	−0.20	−0.15	**−0.90**	−0.18	−0.26
pH	**0.85**	−0.22	0.09	0.35	0.08	0.21	**−0.76**	0.25	0.12	**−0.88**	0.15	0.10	0.31	**−0.83**
Organic matter content	**−0.40**	−0.34	**0.52**	0.30	0.22	0.32	**0.74**	0.19	**0.45**	**0.50**	**0.38**	0.34	0.29	**0.55**
Electrical conductivity	0.02	−0.09	**0.78**	0.13	0.20	**0.75**	0.12	0.31	0.14	0.07	**0.73**	**0.68**	0.02	−0.07
Sand	0.06	**−0.92**	0.02	**0.91**	-0.07	0.03	0.04	0.14	**0.90**	−0.14	−0.04	−0.01	**0.91**	0.00
Silt	0.01	**0.89**	−0.19	**−0.88**	−0.09	−0.12	−0.13	−0.14	**−0.91**	0.01	−0.05	−0.20	**−0.86**	−0.03
Clay	−0.28	**0.79**	0.06	**−0.81**	−0.16	0.09	0.33	−0.46	**−0.61**	0.34	0.25	−0.11	**−0.80**	0.21
Variance (%)	31.2	22.4	17.8	26.5	24.4	15.6	14.6	25.6	22.8	18.3	13.9	29.3	25.5	16.9
Total variance (%)	31.2	53.6	71.4	26.5	51.0	66.6	81.2	25.6	48.3	66.6	80.5	29.3	54.7	71.7

Note: “/” stands for undetected concentrations of PTEs. The values in bold mean the main contribution to the PCA components.

**Table 4:** The PCA results of soil environmental factors for *Japonica* rice.

Item	Cd				Cu				Ni			Zn		
	PC1	PC2	PC3	PC4	PC1	PC2	PC3	PC4	PC1	PC2	PC3	PC1	PC2	PC3
EI	**−0.29**	**−0.69**	−0.05	**0.39**	−0.10	**0.44**	**−0.75**	−0.04	0.04	−0.25	**−0.75**	−0.31	−0.22	**−0.62**
Exchangeable	**−0.94**	0.12	0.17	0.15	/	/	/	/	−0.04	0.05	**−0.92**	0.26	0.13	**−0.90**
Fe-Mn bound	**0.94**	0.07	−0.18	−0.06	**−0.88**	0.08	0.24	0.27	**0.77**	−0.48	0.13	**0.93**	−0.13	−0.02
Organic bound	**0.69**	0.04	0.11	0.33	**0.86**	−0.09	−0.10	0.38	**0.89**	0.21	0.13	**0.75**	0.17	0.07
Residual	0.17	**−0.58**	−0.13	**−0.51**	−0.18	0.01	−0.17	**−0.88**	**−0.89**	0.25	0.27	**−0.93**	0.09	0.21
pH	**0.89**	0.18	−0.11	−0.07	0.00	0.26	**0.81**	−0.05	0.15	−0.30	**0.80**	0.03	−0.20	**0.89**
Organic matter	0.05	**0.76**	0.38	0.26	**0.78**	−0.37	0.27	0.19	**0.63**	**0.60**	0.27	**0.60**	**0.61**	0.28
Electrical conductivity	0.02	**0.85**	0.02	0.01	**0.68**	−0.04	0.31	0.17	**0.62**	0.29	0.25	**0.58**	0.30	0.27
Sand	0.05	0.00	0.10	**0.79**	0.04	−0.15	−0.14	**0.61**	0.30	0.21	−0.19	0.21	0.28	−0.05
Silt	0.09	−0.17	**−0.89**	−0.15	−0.21	**0.86**	0.01	−0.12	−0.12	**−0.83**	0.04	−0.03	**−0.87**	0.07
Clay	−0.17	0.09	**0.92**	0.03	0.07	**−0.94**	−0.02	0.08	−0.05	**0.89**	−0.04	−0.15	**0.92**	−0.08
Variance (%)	29.0	19.9	17.3	11.5	26.8	20.5	15.1	14.6	28.2	22.4	21.2	29.2	20.9	20.0
Total variance (%)	29.0	48.8	66.2	77.6	26.8	47.4	62.4	77.0	28.2	50.6	71.7	29.2	50.1	70.1

Note: “/” stands for undetected concentrations of PTEs. The values in bold mean the main contribution to the PCA components.

**Table 5:** Comparison of the coefficients of determination for different simulated models.

Simulated Models	Hybrid Rice				*Japonica* Rice			
	Cd	Cu	Ni	Zn	Cd	Cu	Ni	Zn
Model 1	0.49 **	0.61 **	0.57 **	0.61 **	0.62 **	0.61 **	0.62 **	0.65 **
Model 2	0.72 **	0.83 **	0.76 **	0.78 **	0.81 **	0.76 **	0.70 **	0.78 **
Model 3	0.36 NS	0.55 **	0.54 **	0.54 **	0.40 *	0.64 **	0.55 **	0.59 **
Model 4	0.38 NS	0.55 **	0.52 **	0.53 **	0.38 NS	0.64 **	0.51 **	0.60 **

Note: Model 1 was based on the log-transformed data of PTEs’ fractions and soil physico-chemical properties; Model 2 was based on log-transformed data of PTEs’ fractions and raw data of soil physico-chemical properties; Model 3 was based on raw data of PTEs’ fractions and log-transformed data of soil physico-chemical properties; Model 4 was based on raw data of PTEs’ fractions and soil physico-chemical properties. NS means not significance at α = 0.05 level; * means significance at α = 0.05 level; ** means significance at α = 0.01 level.

**Table 6:** Coefficients of determination (R) of the transfer models for PTEs in soil-rice system.

Factors	Hybrid Rice				*Japonica* Rice			
	Cd	Cu	Ni	Zn	Cd	Cu	Ni	Zn
PTEs’ fractions in soils	0.65 **	0.55 **	0.20 ^NS^	0.57 **	0.50 **	0.31 *	0.65 **	0.60 **
Soil P&C Properties	0.56 **	0.68 **	0.70 **	0.73 **	0.76 **	0.72 **	0.63 **	0.73 **
PTEs’ fractions + P&C Properties	0.72 **	0.83 **	0.76 **	0.78 **	0.81 **	0.76 **	0.70 **	0.78 **

NS indicates that the regression model has not reached the significance at level of 0.05, * indicates a significance at the level of 0.05, ** indicates a significant level of 0.01. Soil P&C Properties means soil physico-chemical properties.

**Table 7:** Coefficients of regression models for the transfer of PTEs in the soil-rice system.

		Exchangeable	Fe-Mn Oxide Bound	Organic Bound	Residual	pH	SOM	EC	Sand	Silt	Clay	Constant
Hybrid rice	Cd	5.99	6.69	−0.83	0.92	−0.39	−0.016	−0.0020	−0.0053	0.0052	−0.039	12.99
	Cu	-	−0.93	0.91	0.84	−0.15	−0.17	−0.0023	0.011	0.013	−0.024	−0.89
	Ni	−0.48	−0.36	−0.011	−6.55	−0.88	−0.25	−0.0027	0.0012	−0.004 6	−0.037	−0.97
	Zn	0.084	−0.18	0.090	0.61	−0.045	0.087	−0.0009	0.008 7	0.012	−0.027	−1.19
*Japonica* Rice	Cd	1.48	1.51	0.22	−0.28	−0.51	−0.19	−0.0008	−0.0083	−0.010	−0.011	4.57
	Cu	-	−0.69	−0.43	−0.95	−0.16	−0.074	−0.0002	0.019	−0.0014	−0.028	−2.76
	Ni	0.15	−0.72	−0.88	−4.27	−0.059	0.007 9	−0.0001	0.064	0.0046	−0.024	−9.51
	Zn	0.058	−1.62	−0.45	−2.46	−0.062	−0.052	−0.0001	−0.016	−0.0097	−0.016	−4.47
